# Supplementary material for: Mayaro Virus Non-Structural Protein 2 Circumvents the Induction of Interferon in Part by Depleting Host Transcription Initiation Factor IIE Subunit 2
Source: Cells. 2021 Dec 12;10(12):3510. doi: 10.3390/cells10123510 (PMC8700540; doi:10.3390/cells10123510)
Supplement: Supplementary file 1 [file cells-10-03510-s001.zip › MAYV-IFN Project Supplementary Materials.pdf]

Supplementary Materials

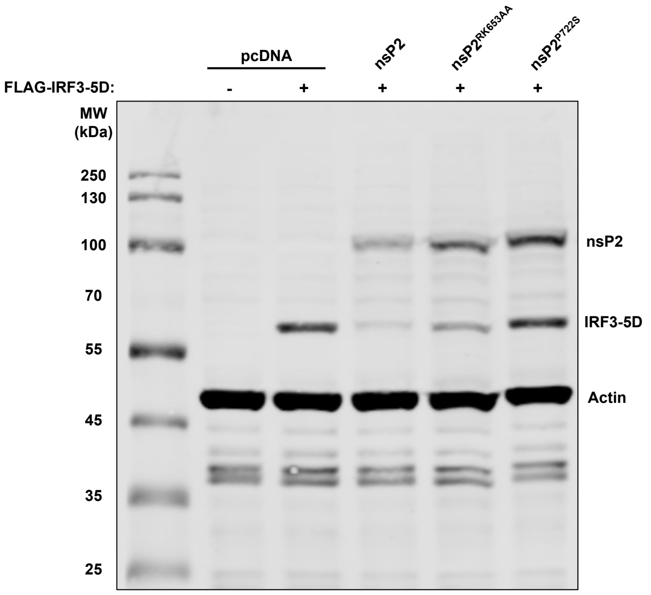

Figure S1. Full blot image from Figure 3E.

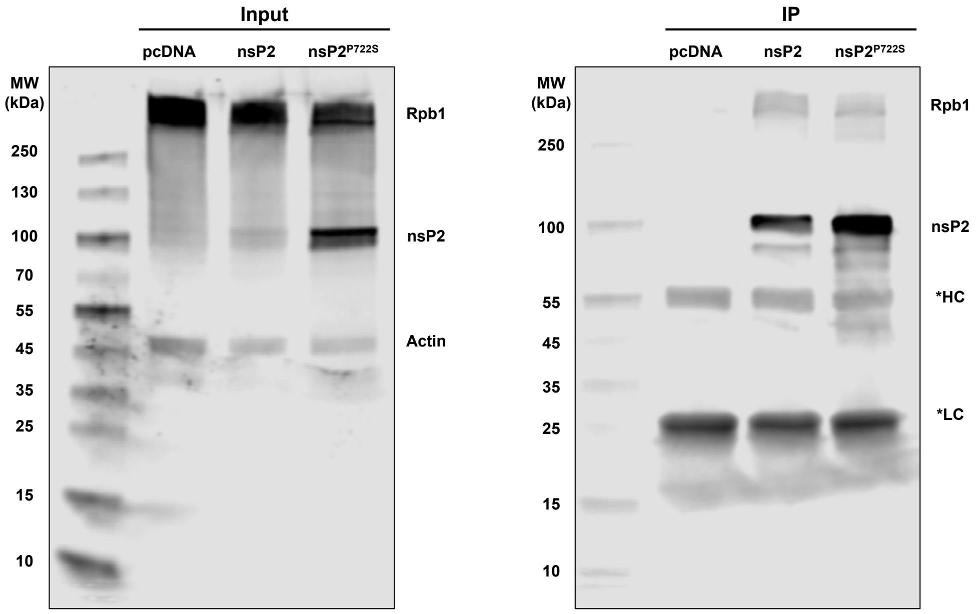

Figure S2. Full blot images from Figure 4B.

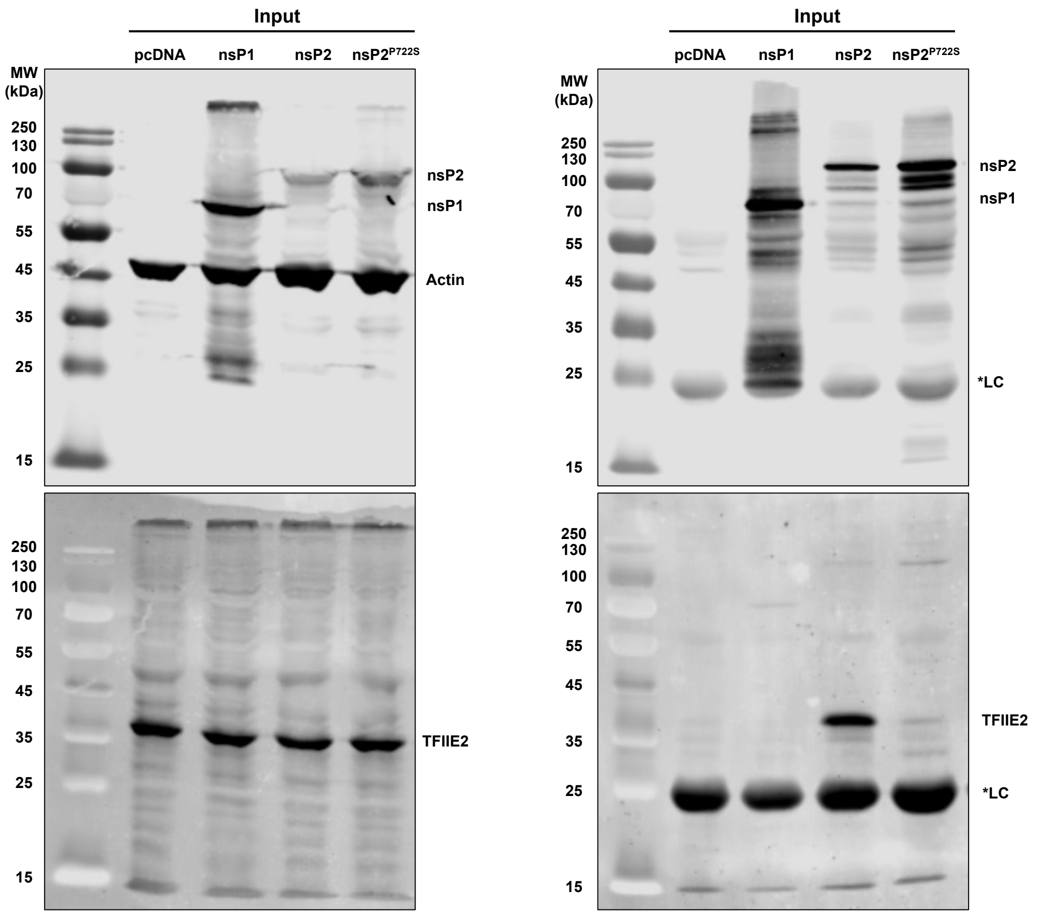

Figure S3. Full blot images from Figure 4C.

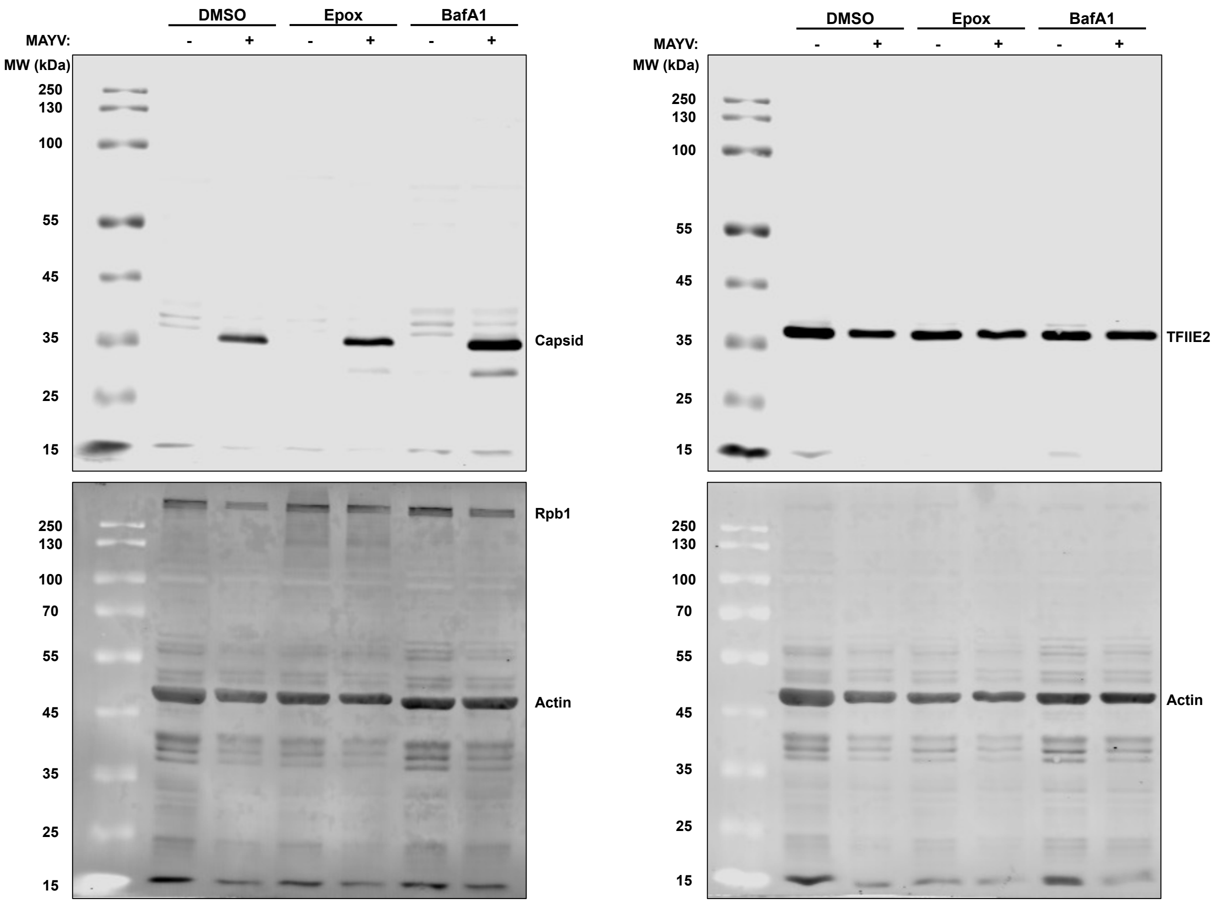

Figure S4. Full blot images from Figure 5E.

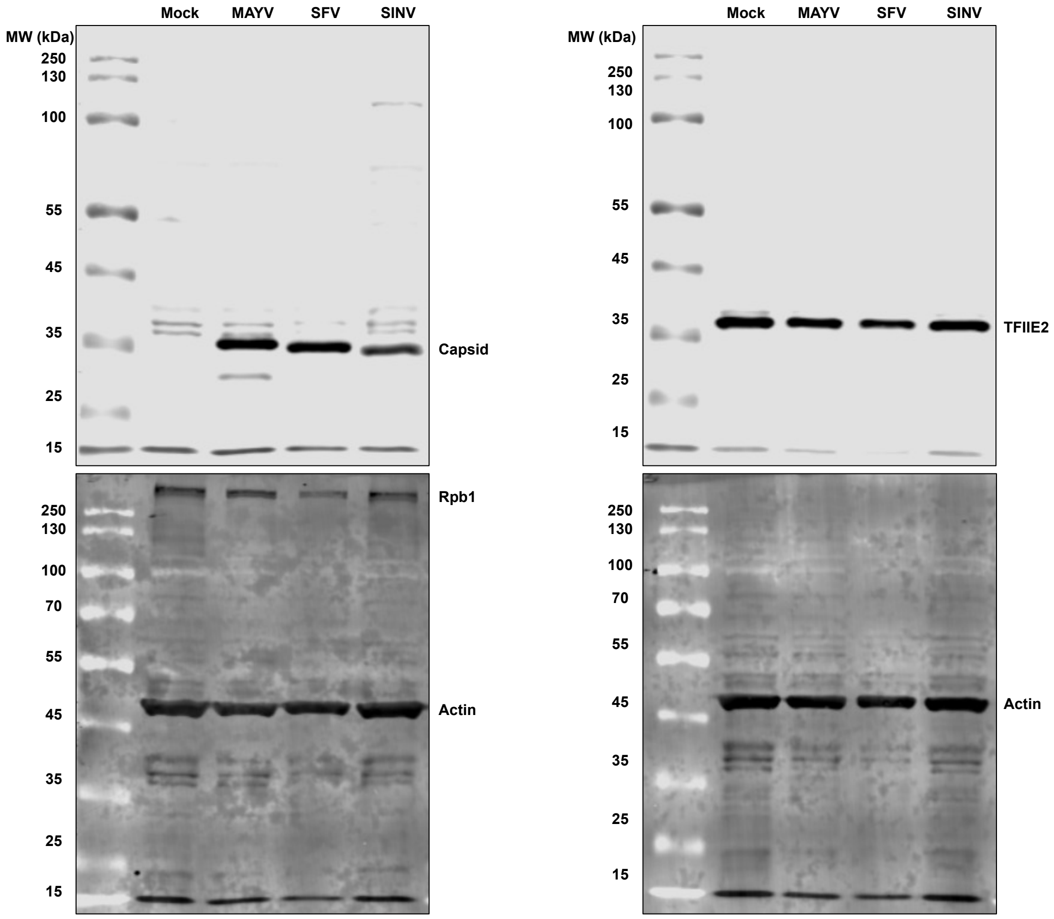

Figure S5. Full blot images of Figure 5H.

Table S1. Primers used for cloning viral proteins.

**PCR Target**	**Primer sequences (5'→3')**
nsP1	Fwd: ATATATGCTAGCATGTCGAAAGTCTTTGTAGATATC<br>Rev: ATATATGGCCGGCCAAACTCCAACTCTTCCACATCCAC
nsP2	Fwd: ATATATGCTAGCATGCGAGCGGGAGCCGGTGTTGTCGAGA<br>Rev: ATATATGGCCGGCCGCAACCAGCTGCCTGCAGCACTGT
nsP3	Fwd: ATATATGCTAGCATGGCTCCAGTGTATGCCGTTAAAAGG<br>Rev: ATATATGGCCGGCCTCAAGATGAATTACTAATGGTTTC
nsP4	Fwd: ATATATGCTAGCATGCTAGGCCGTGCGGGGGCCTATATT<br>Rvev: ATATATGGCCGGCCTTTAGGACCGCCGTACAGATGTAT
E3-E2	Fwd: ATATATGCTAGCATGGCGGCCTCGACAGTGACAGCTATG<br>Rev: ATATATGGCCGGCCTGCATGTGCTTTCGGTGCGCAACA
C	Fwd:<br>GCTGGCTAGCATGGACTACAAAGACCATGACGGTGATTATAAAGATCATGACATCGA<br>CTACAAGGATGACGATGACAAGGGCGGGAGCGGCGGGATGGACTTCCTACCAACTC<br>AAGTGTT<br>Rev: GTTTAAACTTAAGCTTGGATCCTA
6K-E1	Fwd: ATATATGCTAGCATGGCAACAAATGTCTGACACCATATG<br>Rev: ATATATGGCCGGCCCCTTCTCAAAGTTATGCAAGTAAC
nsP2$^{\Delta protease}$	Fwd: CAAGCTGGCTAGCATGCGAGCGGG<br>Rev: CCGCCGGGCCGGCCCACATCCAAAGGAGCTGCTTCCTG
nsP2$^{\Delta helicase}$	Fwd: AGCTGGCTAGCATGTTCCAGAATAAAGCTAAAGTGTGC<br>Rev: CGCCGGGCCGGCCGCAACCAGCTG
nsP2^{K197N}	Fwd: CAAGCTGGCTAGCATGCGAGCGGG<br>Rev: TTCCAAAACGGGTACCAGGCATTT<br>Fusion fwd:<br>GTCTTGGGCGTGCCCGGATCTGGAAATTCAGGTATAATCAAGAGCC TGGTT<br>Fusion rev:<br>AACCAGGCTCTTGATTATACCTGAATTTCCAGATCCGGGCACGCCCA AGAC
nsP2^{C478A}	Fwd: CAAGCTGGCTAGCATGCGAGCGGG<br>Rev: AAAACGGGTACCAGGCATTTGGCCCAGGCCACTTTAGCTTTATTCTGGAAC
nsP2^{RK653AA}	Fwd: AAATGCCTGGTACCCGTTTTGGAA<br>Rev: CGCCGGGCCGGCCGCAACCAGCTG<br>Fusion fwd:<br>GAGTACAACCTCATCTTGCCGAGGGCAGCGGTGACGTGGATTGCTCCGCCGACT<br>Fusion rev:<br>AGTCGGCGGAGCAATCCACGTCACCGCTGCCCTCGGCAAGATGAGGTTGTACTC
nsP2^{P722S}	Fwd: AAATGCCTGGTACCCGTTTTGGAA<br>Rev: CGCCGGGCCGGCCGCAACCAGCTG<br>Fusion fwd: GCACTTTACCTACTGAAATCTGGGGGAAGCCTCCTTTTGAG<br>Fusion rev: CTCAAAAGGAGGCTTCCCCCAGATTTCAGTAGGTAAAGTGC

Table S2. Primers used for qRT-PCR.

**Target Gene**	**Primer sequences (5'→3')**
MAYV nsP1	Fwd: TTCCGAACCAAGTGGGATTC<br>Rev: CACTTTACGTAYGGKGATGG
SeV HN	Fwd: AAAATTACATGGCTAGGAGGGAAAC<br>Rev: GTGAATGGAATGGTTGTGACTCTTA
ACTB	Fwd: CCTGGCACCCAGCACAAT<br>Rev: GCCGATCCACACGGAGTACT
ifnb	Fwd: TAGCACTGGCTGGAATGAGA<br>Rev: TCCTTGGCCTTCAGGTAATG
ifnl2	Fwd: AGTTCCGGGCCTGTATCCAG<br>Rev: GAGCCGGTACAGCCAATGGT
GAPDH	Fwd: ACAGTCAGCCGCATCTTCTT<br>Rev: GTTAAAAGCAGCCCTGGTGA
Tuba1a	Fwd: GCAACAACCTCTCCTCTTCG<br>Rev: GAATCATCTCCTCCCCCAAT

Table S3. MiST analysis-based scoring of nsP2 interacting cellular proteins.

Table S4. GO enrichment analysis of nsP2 interacting cellular proteins.
